# Multi‐Omics Landscape of Paraspinal Muscles in Spinal Muscular Atrophy With Scoliosis

**DOI:** 10.1111/jcmm.71279

**Published:** 2026-07-05

**Authors:** Zhen Wang, Junduo Zhao, Xu'an Huang, Weiyun Chen, Jianxiong Shen

**Affiliations:** ^1^ Department of Orthopedics Peking Union Medical College Hospital, Peking Union Medical College and Chinese Academy of Medical Sciences Beijing People's Republic of China; ^2^ Department of Orthopedics The First Affiliated Hospital of Nanjing Medical University Nanjing People's Republic of China; ^3^ Department of Anesthesiology, State Key Laboratory of Complex Severe and Rare Diseases Peking Union Medical College Hospital, Peking Union Medical College, Chinese Academy of Medical Science Beijing People's Republic of China

**Keywords:** asymmetric disparities, integrated multi‐omics, metabolic reprogramming, multifidus, spinal muscular atrophy

## Abstract

Most spinal muscular atrophy (SMA) patients develop severe scoliosis by late adolescence. Given that the paraspinal muscles—particularly the multifidus—are indispensable for maintaining spinal stability, their site‐specific multi‐omics characteristics in SMA remain insufficiently defined. Herein, integrated multi‐omics sequencing was performed on bilateral multifidus samples from SMA patients and surgical controls. We identified 5219 differentially expressed genes, 1063 differentially expressed proteins and 370 differential metabolites between the control and SMA, showing significant enrichment in glucose and amino acid metabolism pathways, specifically key steps of glycolysis/gluconeogenesis. Key enzymes in the glycolytic process such as PFKM, ENO3 and PKM1 were markedly downregulated. Notably, a comparative analysis of the bilateral paraspinal muscles in SMA revealed asymmetrical metabolic signatures in carbohydrate and amino acid processing between the concave and convex sides. Key regulatory enzymes exhibited significant differential expression: PYGL, a central driver of starch and sucrose metabolism; creatine kinase, involved in arginine and proline metabolism; and PGAM2, a key mediator of glycine, serine, and threonine metabolism. These metabolic signatures indicate a complex metabolic reprogramming in the multifidus, where asymmetric disparities point to the influence of mechanical loading, while systemic dysregulation aligns with the effects of SMN depletion.

**Abbreviations**
ALS: amyotrophic lateral sclerosis
AMPK: adenosine 5′‐monophosphate‐activated protein kinase
BP: biological process
cAMP: cyclic adenosine 5′‐monophosphate
CC: cellular component
C_L: muscles collected from the left side in control individuals
C_R: muscles collected from the right side in control individuals
CS: congenital scoliosis
DDA: data‐dependent acquisition
DEG: differentially expressed gene
DEP: differentially expressed protein
DIA: data‐independent acquisition
DM: differential metabolite
DMD: Duchenne muscular dystrophy
DO: disease ontology
GO: gene ontology
IS: idiopathic scoliosis
KEGG: Kyoto Encyclopedia of Genes and Genomes
LC: liquid chromatography
LDH: lumbar disc herniation
LS: lumbar spondylolysis
MF: molecular function
MRS: magnetic resonance spectroscopy
MS: mass spectrometry
NADPH: nicotinamide adenine dinucleotide phosphate hydrogen
PPI: protein–protein interaction
SMA: spinal muscular atrophy
SMA_A: muscles obtained from the concavity in SMA
SMA_T: muscles obtained from the convexity in SMA
SMN: survival motor neuron protein
UHPLC: ultra‐high performance liquid chromatography

## 1 Introduction

Spinal muscular atrophy (SMA) is a hereditary neuromuscular disorder caused by the homozygous deletion or mutation of the *SMN1* gene. This defect reduces survival motor neuron (SMN) protein levels, leading to progressive degeneration of spinal cord α‐motor neurons and subsequent symmetric flaccid paralysis of the trunk and limbs. Historically, SMA was the leading genetic cause of infant mortality, with a neonatal incidence of approximately 1/12000 [1]. Over the past decade, however, the therapeutic landscape has been transformed. Disease‐modifying therapies—including antisense oligonucleotides, gene augmentation, and small‐molecule splicing modifiers—have shifted care from palliation to proactive intervention. These treatments can slow disease progression and, when given pre‐symptomatically, markedly mitigate the consequences of SMN deficiency [2]. As the “infant mortality” label fades, research focus has shifted toward characterizing long‐term muscle health and structural stability in SMA survivors, which has emerged as a key scientific priority.

Traditional views categorized muscular atrophy in SMA as purely neurogenic. However, Cifuentes‐Diaz et al. [3] demonstrated that selective deletion of murine *Smn* in skeletal muscles evoked primary muscular dystrophy, while muscle‐specific restoration of SMN alleviated the phenotype [4], redefining SMA as a multi‐system disorder. To date, integrated omics approaches have been implemented to elucidate muscle pathogenesis in SMA mouse models [5], clinical specimens [6] and iPSC‐derived myotubes [7]. Notably, muscle MRI in SMA demonstrates a highly selective involvement pattern, featuring a proximal‐to‐distal gradient and compartmental disparities, with the adductor longus and gracilis consistently spared [8]. Thus, muscle‐specific omics profiling may provide critical insights into these distinct pathological manifestations in SMA. Besides motor and respiratory dysfunction, progressive scoliosis constitutes a major complication in SMA [9], with surgical rates of 84% in Type 2 and 40% in Type 3a patients [10]. As a core deep paraspinal muscle, the multifidus provides segmental stability to the spine. In idiopathic scoliosis (IS), the concave multifidus is severely affected, and its degeneration correlates with curve severity [11]. Nevertheless, it remains unclear whether the bilateral multifidus presents asymmetric multi‐omics disturbances in SMA‐associated scoliosis.

This study aimed to profile the transcriptomic, proteomic, and metabolic signatures of paraspinal muscles in SMA. Using integrated multi‐omics, we explored molecular abnormalities and asymmetries between concave and convex multifidus, providing new insights into muscle pathogenesis in SMA‐related scoliosis.

## 2 Methods

### 2.1 Subject

**FIGURE 1:**
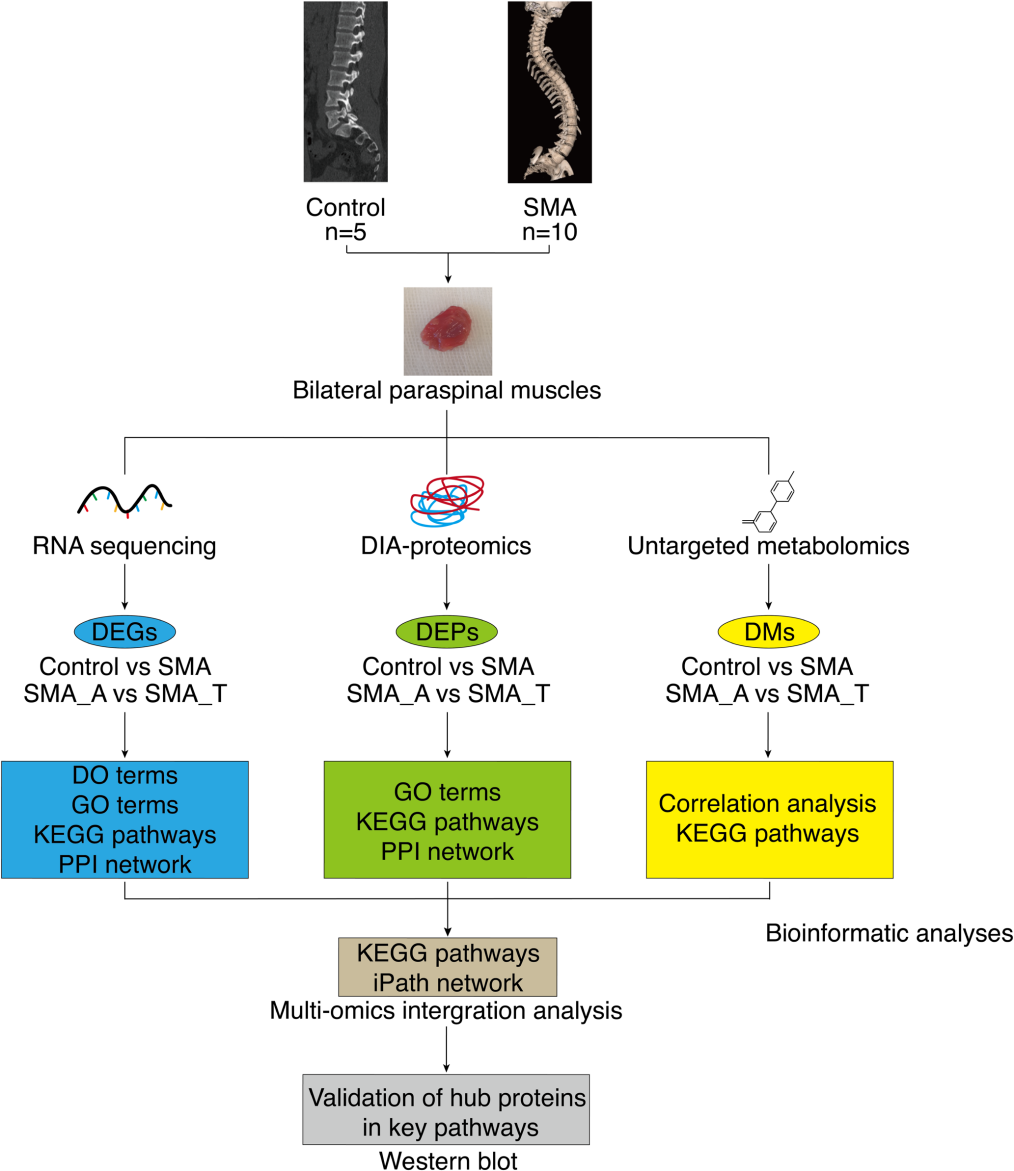
Study design and workflow. Bilateral paraspinal muscles were intraoperatively harvested from 10 patients with SMA and 5 control individuals. Integrated multi‐omics analysis was performed, encompassing RNA sequencing, DIA‐based proteomics, and untargeted metabolomics for the extraction and profiling of mRNAs, proteins, and metabolites, respectively. DEGs, DEPs, and DMs were identified through inter‐group (SMA vs. control) and intra‐group (SMA_A vs. SMA_T) comparisons. Bioinformatic characterization included DO, GO, KEGG pathway enrichment, and PPI network analyses. Cross‐omics integration was implemented by mapping DEGs, DEPs, and DMs to KEGG pathways and the iPath network. Finally, hub proteins within pivotal pathways were validated via immunoblotting. SMA: Spinal muscular atrophy; DIA: Data‐independent acquisition; DEG: Differentially expressed gene; DEP: Differentially expressed protein; DM: Differential metabolite; SMA_A: Concave side; SMA_T: Convex side; DO: Disease ontology; GO: Gene ontology; KEGG: Kyoto Encyclopedia of Genes and Genomes; PPI: Protein–protein interaction; iPath: Interactive pathways explorer.

SMA patients admitted to Peking Union Medical College Hospital between May 2021 and April 2023 were enrolled. Inclusion criteria for the SMA group were: (1) genetically confirmed diagnosis; (2) undergoing primary posterior spinal instrumentation; (3) age < 30 years at surgery. Inclusion criteria for the control group were: (1) lumbar diseases without spinal deformities; (2) age < 30 years at surgery; (3) symptoms duration < 6 months with no acute exacerbations or glucocorticoid use in the prior month; (4) a Goutallier grade ≤ 1 on preoperative MRI. Importantly, this control cohort provides a biomechanically neutral baseline, establishing a near‐normal, symmetric spinal reference that minimizes confounding factors and allows precise delineation of the paraspinal muscle molecular profiles in SMA. Participant characteristics and study workflow are summarized in Table 1 and Figure 1, respectively.

**TABLE 1:** Clinical and demographic characteristics of the SMA patients and control individuals.

Group	Age (y)	Gender	Diagnosis	Application
C1	12	F	LS (L5)	Multi‐omics sequencing
C2	14	M	LS (L5)	Multi‐omics sequencing
C3	20	F	LDH (L5/S1)	Multi‐omics sequencing
C4	23	F	LDH (L5/S1)	Multi‐omics sequencing
C5	21	M	LS (L5)	Multi‐omics sequencing
C6	29	F	LDH (L5/S1)	Immunoblot (SMA vs. C)
C7	24	M	LDH (L4/5)	Immunoblot (SMA vs. C)
C8	24	M	LDH (L4/5、L5/S1)	Immunoblot (SMA vs. C)
SMA1	21	M	SMA (Type 3)	Multi‐omics sequencing
SMA2	26	M	SMA (Type 2)	Multi‐omics sequencing
SMA3	13	F	SMA (Type 3)	Multi‐omics sequencing
SMA4	15	F	SMA (Type 3)	Multi‐omics sequencing
SMA5	13	F	SMA (Type 2)	Multi‐omics sequencing
SMA6	12	F	SMA (Type 2)	Multi‐omics sequencing
SMA7	16	F	SMA (Type 2)	Multi‐omics sequencing
SMA8	14	M	SMA (Type 2)	Multi‐omics sequencing
SMA9	13	F	SMA (Type 2)	Multi‐omics sequencing
SMA10	11	F	SMA (Type 2)	Multi‐omics sequencing
SMA11	28	M	SMA (Type 2)	Immunoblot (SMA vs. C and SMA_A vs. SMA_T)
SMA12	20	F	SMA (Type 3)	Immunoblot (SMA vs. C and SMA_A vs. SMA_T)
SMA13	23	F	SMA (Type 2)	Immunoblot (SMA vs. C and SMA_A vs. SMA_T)
SMA14	12	F	SMA (Type 2)	Immunoblot (SMA_A vs. SMA_T)
SMA15	12	F	SMA (Type 2)	Immunoblot (SMA_A vs. SMA_T)

Abbreviations: LDH, lumbar disc herniation; LS, lumbar spondylolysis; SMA, spinal muscular atrophy.

### 2.2 Sample Acquisition, Processing, and Preservation

Bilateral multifidus samples were harvested intraoperatively from the apex of the major curve in SMA patients and at the L5 level in controls. Each sample was divided into three aliquots for multi‐omics profiling: (1) Transcriptomics: immersed in RNAlater (Beyotime Biotechnology, China) and stored at −80°C; (2) Data‐Independent Acquisition (DIA)‐Mass Spectrometry (MS) proteomics: preserved in ALLPROTECT stabilization solution (Beyotime Biotechnology) and stored at −80°C; (3) Liquid Chromatography (LC)‐MS metabolomics: flash‐frozen in liquid nitrogen for 15 min, then stored at −80°C. All samples were analysed without repeated freeze–thaw cycles.

### 2.3 Experimental Grouping

Paraspinal muscles from SMA patients and controls were assigned to SMA and control groups for multi‐omics comparison. Within the SMA group, concave and convex specimens were designated as SMA_A and SMA_T subgroups, respectively, to explore asymmetric muscle differences. Control specimens were stratified by side into right (C_R) and left (C_L) subgroups.

### 2.4 Transcriptomic Analysis

Total RNA was extracted using the TRIzol method, with quality, integrity, and purity assessed via an Agilent 2100 Bioanalyzer (Agilent Technologies, USA). mRNA was enriched by oligo (dT) magnetic beads for library construction. Library concentration was quantified with a Qubit 2.0 Fluorometer (Thermo Fisher Scientific, USA) and verified by qPCR, followed by sequencing on the Illumina NovaSeq 6000 platform (Illumina, USA).

Raw reads were filtered to obtain clean data and aligned to the reference genome using HISAT2 (v2.0.5). Gene expression was quantified with FeatureCounts (v1.5.0‐p3). Differential expression analysis was performed using DESeq2 (v1.20.0), with |log_2_(fold change)| > 1 and raw *p* < 0.05 set as thresholds for differentially expressed genes (DEGs) for both the SMA vs. control comparison and the paired SMA_A vs. SMA_T comparison. Functional enrichment analyses, including Gene Ontology (GO), Disease Ontology (DO), and Kyoto Encyclopedia of Genes and Genomes (KEGG) pathways, were performed with clusterProfiler (v3.8.1). Protein–protein interaction (PPI) networks were constructed using the STRING database.

### 2.5 DIA‐Based Quantitative Proteomics Profiling

Tissues were lysed in SDT buffer, sonicated for 5 min, and centrifuged at 12000 g for 15 min. Supernatants were alkylated with iodoacetamide for 1 h and incubated with acetone for 2 h to precipitate proteins and recentrifuged. Protein pellets were redissolved, quantified, digested with trypsin, desalted, and lyophilized. Peptides were fractionated using an L‐3000 HPLC system (Arc Scientific, USA) and analysed via EASY‐nLC 1200 UHPLC coupled with a Q Exactive HF‐X mass spectrometer (Thermo Fisher Scientific).

Data‐Dependent Acquisition (DDA) data were searched against the UniProt human database (2023‐03‐13, 2,07,393 sequences) using Spectronaut Pulsar (Biognosys, Schlieren, Switzerland) with the following parameters: mass tolerance for precursor ion, 10 ppm; mass tolerance for product ion, 0.02 Da; fixed modification, carbamidomethyl; dynamic modification, oxidation of methionine; N‐terminal modification, acetylation; maximum of missed cleavage sites, 2. Retrieval results were filtered with a peptide‐spectrum match confidence > 99% and false discovery rate < 1% for peptides and proteins.

DIA data were quantified against the DDA spectral library. Identified proteins were annotated using GO, KEGG, COG/KOG, and Pfam databases. Differentially expressed proteins (DEPs) were identified using thresholds of |log_2_(fold change)| > 1 and raw *p* < 0.05 for both the SMA vs. control comparison and the paired SMA_A vs. SMA_T comparison, followed by GO/KEGG enrichment and PPI network analysis.

### 2.6 Untargeted LC–MS/MS Metabolomics Profiling

Tissues were homogenized in liquid nitrogen and extracted with 80% methanol. After a 5‐min ice bath and centrifugation at 15000 g for 20 min at 4°C, the supernatants were diluted to 53% methanol with LC–MS grade water. Pooled quality control samples and 53% methanol blanks were prepared accordingly. Metabolite profiling was performed using a Vanquish UHPLC system coupled with an Orbitrap Q Exactive HF spectrometer (Thermo Fisher Scientific).

Raw data were processed with Compound Discoverer 3.1 for peak alignment, extraction (mass tolerance 5 ppm, intensity tolerance 30%) and normalization. Metabolites were identified using mzCloud, mzVault and Masslist databases, and annotated via KEGG, HMDB, and LIPIDMaps. Differential metabolites (DMs) were defined by VIP > 1 (from PLS‐DA), Fold Change > 1.2 or < 0.83, and raw *p* < 0.05 for both the SMA vs. control comparison and the paired SMA_A vs. SMA_T comparison, followed by KEGG pathway enrichment analysis.

### 2.7 Integrated Multi‐Omics Analysis

Transcriptomic, proteomic and metabolomic datasets were integrated by mapping DEGs, DEPs and DMs to KEGG pathways and metabolic pathways in Interactive Pathways Explorer (iPath; https://pathways.embl.de/). Pathway enrichment was determined by comparing the ratio of differential features to total differential entities against the ratio of all annotated features in the pathway to total features across all pathways. For transcriptomic analysis, for instance, enrichment was defined as x/*n* > y/N (x: DEGs in a specific pathway; n: total DEGs; y: all genes annotated to the pathway; N: total genes across all pathways), with the same logic applied to proteomic and metabolomic analyses.

### 2.8 Western Blotting

Total proteins were extracted from tissues using RIPA lysis buffer (Beyotime) supplemented with 1 mM PMSF and 1 mM phosphatase inhibitor cocktail. Protein concentrations were quantified by BCA assay (Beyotime). Equal amounts of protein were separated by 10% SDS‐PAGE, transferred to PVDF membranes (Merck Millipore), blocked, and incubated with primary antibodies (Table 2) followed by HRP‐conjugated secondary antibodies. Signals were detected using the Tanon 5200 Multi Chemiluminescence Imaging System, and protein expression was normalized to β‐tubulin.

**TABLE 2:** Primary antibodies used for western blot analysis.

Protein	Brand	Cat No.
ENO3	Proteintech	68147‐1‐Ig
PKM1	Proteintech	15821‐1‐AP
PFKM	Proteintech	55028‐1‐AP
MDH2	Proteintech	15462‐1‐AP
PGAM2	Proteintech	15550‐1‐AP
PYGL	Proteintech	66769‐1‐Ig
CKM	Proteintech	60177‐1‐Ig

## 3 Results

### 3.1 Patient Characteristics for Multi‐Omics Sequencing

The control cohort comprised three patients with lumbar spondylolysis and two patients with lumbar disc herniation. The SMA group included 10 patients (7 type 2, 3 type 3). No significant differences were observed in age or sex ratio between groups (Table 3).

**TABLE 3:** Comparison of clinical and demographic characteristics between the control and SMA groups.

	Control	SMA	*p*
Age (year)^a^	18.0 ± 4.7	15.4 ± 4.6	0.329
Female no. (%)^b^	3 (60%)	7 (70%)	1.000

Abbreviation: SMA, spinal muscular atrophy.

^a^ Continuous variables are presented as mean ± standard deviation (SD) and compared using the independent *t*‐test.

^b^ Categorical variables are presented as percentages and compared using the chi‐square test.

### 3.2 Transcriptomic Landscape of Paraspinal Muscles in SMA

**FIGURE 2:**
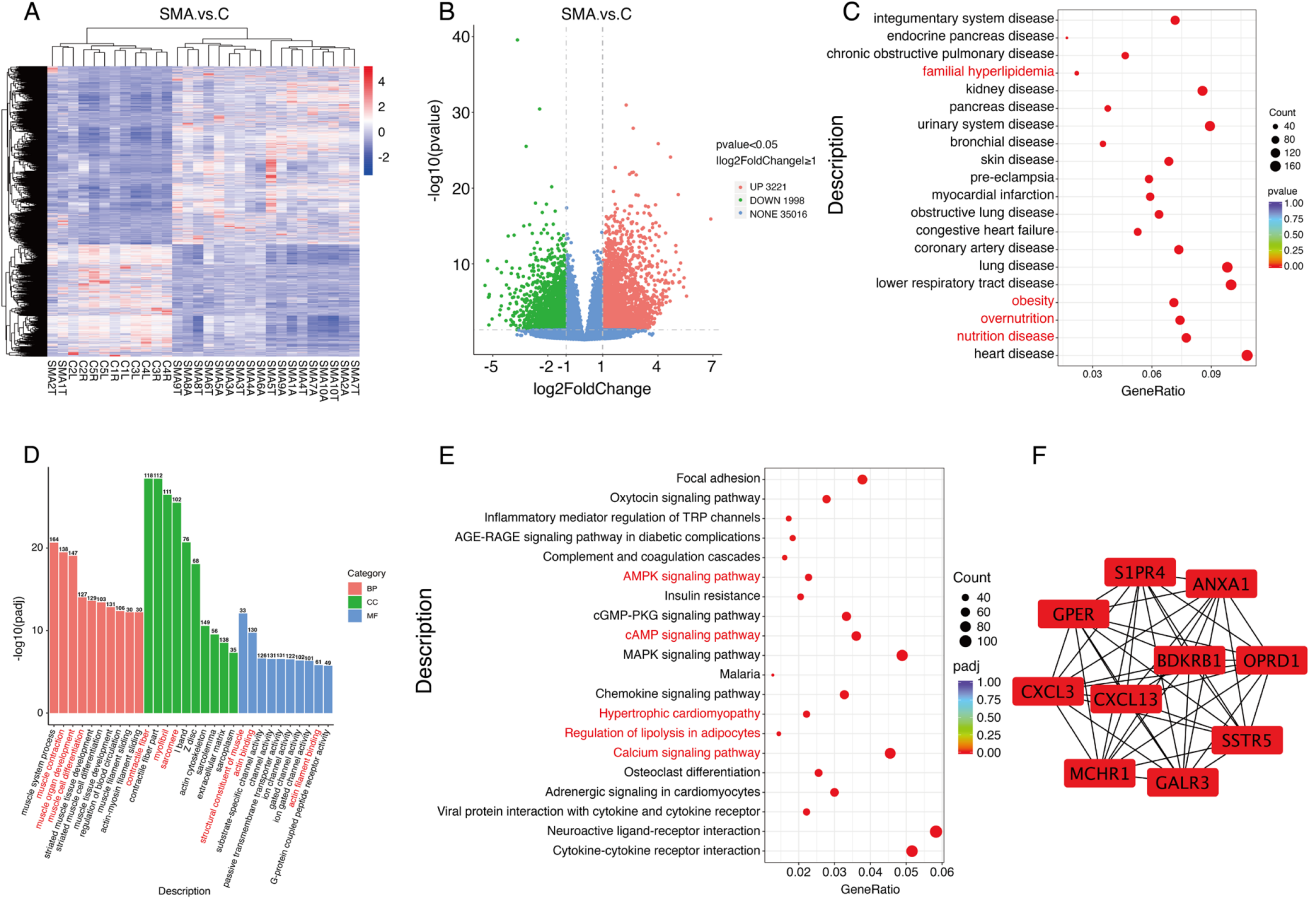
Transcriptomic profiling of SMA paraspinal muscles. (A, B) Heatmap and volcano plot displaying DEGs between the SMA and control groups. (C) Bubble plot of enriched DO terms for DEGs. (D) Histogram showing GO functional enrichment of DEGs across biological processes, cellular components, and molecular functions. (E) Bubble plot of significantly enriched KEGG pathways for DEGs. (F) Top 10 hub genes identified from the PPI network of DEGs. SMA: Spinal muscular atrophy; DEG: Differentially expressed gene; DO: Disease ontology; GO: Gene ontology; KEGG: Kyoto Encyclopedia of Genes and Genomes; PPI: Protein–protein interaction.

Comparison between the SMA and control groups identified 5219 DEGs, including 3221 upregulated and 1998 downregulated genes in SMA (Figure 2A,B). These DEGs were enriched in 153 DO terms, such as nutrition disease, overnutrition and obesity (Figure 2C). Notably, 5 terms were associated with muscular disorders, including myopathy, muscle tissue disease, muscular disease, myasthenia gravis and neuromuscular junction disease. GO enrichment analysis showed that 9 of the top 10 biological processes (BP) were muscle‐related (e.g., muscle contraction, organ development, cell differentiation), 9 of the top 10 cellular components (CC) were myocyte structural compartments (e.g., contractile fibres, myofibrils, sarcomeres), and 3 of the top 10 molecular functions (MF) were muscle‐associated (structural constituent of muscle, actin binding, actin filament binding; Figure 2D). KEGG analysis identified 56 significantly enriched pathways, including calcium signalling, cAMP, cGMP‐PKG, and AMPK signalling pathways (Figure 2E). PPI network analysis revealed the top 10 hub genes: *CXCL13*, *BDKRB1*, *S1PR4*, *GPER*, *CXCL3*, *MCHR1*, *GALR3*, *SSTR5*, *OPRD1*, and *ANXA1* (Figure 2F).

### 3.3 Transcriptomic Divergence Between Bilateral Paraspinal Muscles in SMA

**FIGURE 3:**
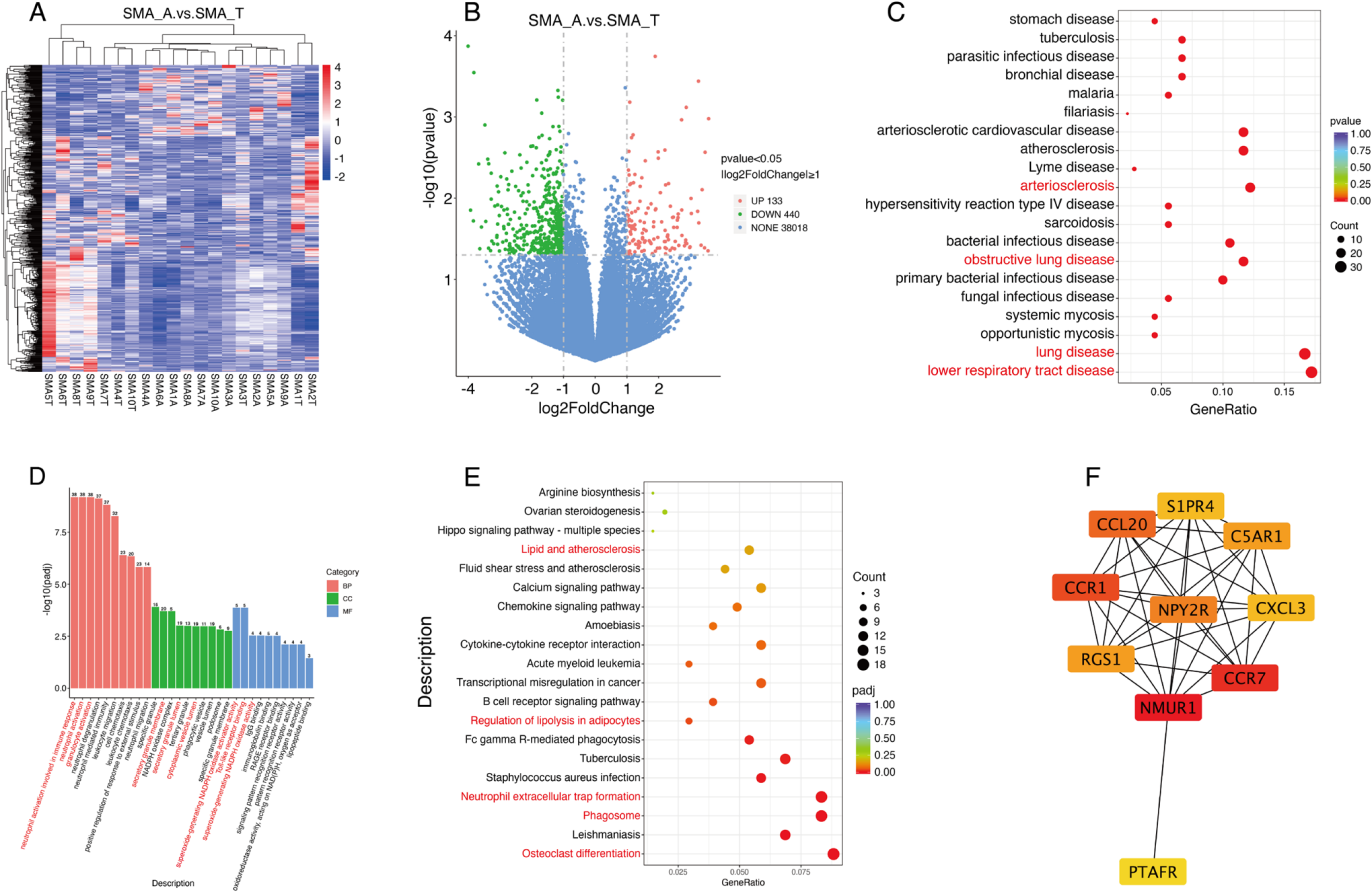
Transcriptomic comparison between concave and convex paraspinal muscles in SMA. (A, B) Heatmap and volcano plot of DEGs between the concave (SMA_A) and convex (SMA_T) sides. (C) Bubble plot showing enriched DO terms for these DEGs. (D) Histogram of GO functional enrichment for DEGs across biological processes, cellular components, and molecular functions. (E) Bubble plot of significantly enriched KEGG pathways. (F) Top 10 hub genes identified from the PPI network of DEGs between the two sides. SMA: Spinal muscular atrophy; SMA_A: Concave side; SMA_T: Convex side; DEG: Differentially expressed gene; DO: Disease ontology; GO: Gene ontology; KEGG: Kyoto Encyclopedia of Genes and Genomes; PPI: Protein–protein interaction.

A comparison of SMA_A versus SMA_T revealed 573 DEGs: 133 upregulated on the concave side and 440 on the convex side (Figure 3A,B). DO enrichment showed associations with lower respiratory tract disease, lung disease, obstructive lung disease, and arteriosclerosis (Figure 3C). GO analysis indicated that DEGs were enriched in immune‐ and inflammation‐related BP terms, including neutrophil activation and granulocyte activation. CC terms mainly involved secretory granule membrane, secretory granule lumen, and cytoplasmic vesicle lumen, while MF terms included superoxide‐generating nicotinamide adenine dinucleotide phosphate (NADPH) oxidase activator activity, Toll‐like receptor binding, and superoxide‐generating NADPH oxidase activity (Figure 3D). KEGG pathway analysis confirmed enrichment in osteoclast differentiation, phagosome, and neutrophil extracellular trap formation (Figure 3E). The top 10 hub genes from the PPI network were *NMUR1*, *NPY2R*, *CCR7*, *CCR1*, *CCL20*, *RGS1*, *PTAFR*, *CXCL3*, *C5AR1*, and *S1PR4* (Figure 3F).

### 3.4 Proteomic Landscape of Paraspinal Muscles in SMA

**FIGURE 4:**
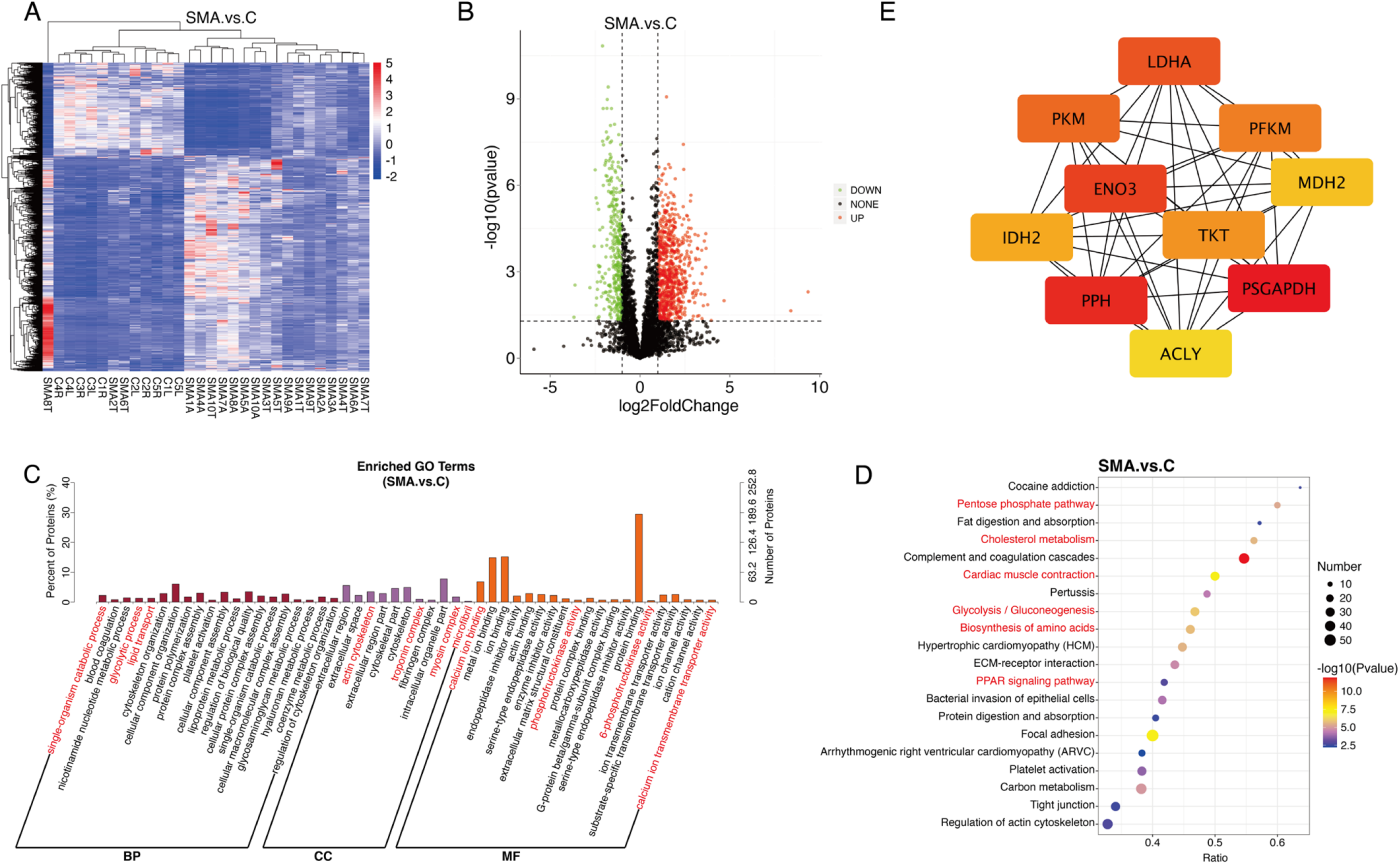
Proteomic profiling of paraspinal muscles in SMA. (A, B) Heatmap and volcano plot of DEPs between the SMA and control groups. (C) Histogram showing GO functional enrichment of DEPs across biological processes, cellular components, and molecular functions. (D) Bubble plot of significantly enriched KEGG pathways for DEPs. (E) Top 10 hub proteins identified from the PPI network of DEPs. SMA: Spinal muscular atrophy; DEP: Differentially expressed protein; GO: Gene ontology; KEGG: Kyoto Encyclopedia of Genes and Genomes; PPI: Protein–protein interaction.

Quantitative analysis identified 1063 DEPs between the SMA and control groups, with 733 upregulated and 330 downregulated in SMA (Figure 4A,B). GO enrichment revealed that these DEPs were mainly involved in BP terms including glycolytic process, lipid transport, and single‐organism carbohydrate metabolic process; CC terms including actin cytoskeleton, troponin complex, myosin complex, and microfibril; and MF terms including calcium ion binding, calcium ion transmembrane transporter activity, phosphofructokinase activity, and 6‐phosphofructokinase activity (Figure 4C). KEGG analysis showed DEPs enriched in the pentose phosphate pathway, cardiac muscle contraction, glycolysis/gluconeogenesis, and biosynthesis of amino acids (Figure 4D). The top 10 hub proteins from PPI network analysis were phosphopyruvate hydratase (PPH), peptidyl‐cysteine S‐nitrosylase glyceraldehyde‐3‐phosphate dehydrogenase (PSGAPDH), enolase 3 (ENO3), pyruvate kinase M1/2 (PKM), phosphofructokinase muscle (PFKM), transketolase (TKT), isocitrate dehydrogenase 2 (IDH2), malate dehydrogenase 2 (MDH2), lactate dehydrogenase A (LDHA), and ATP citrate lyase (ACLY) (Figure 4E).

### 3.5 Proteomic Divergence Between Bilateral Paraspinal Muscles in SMA

**FIGURE 5:**
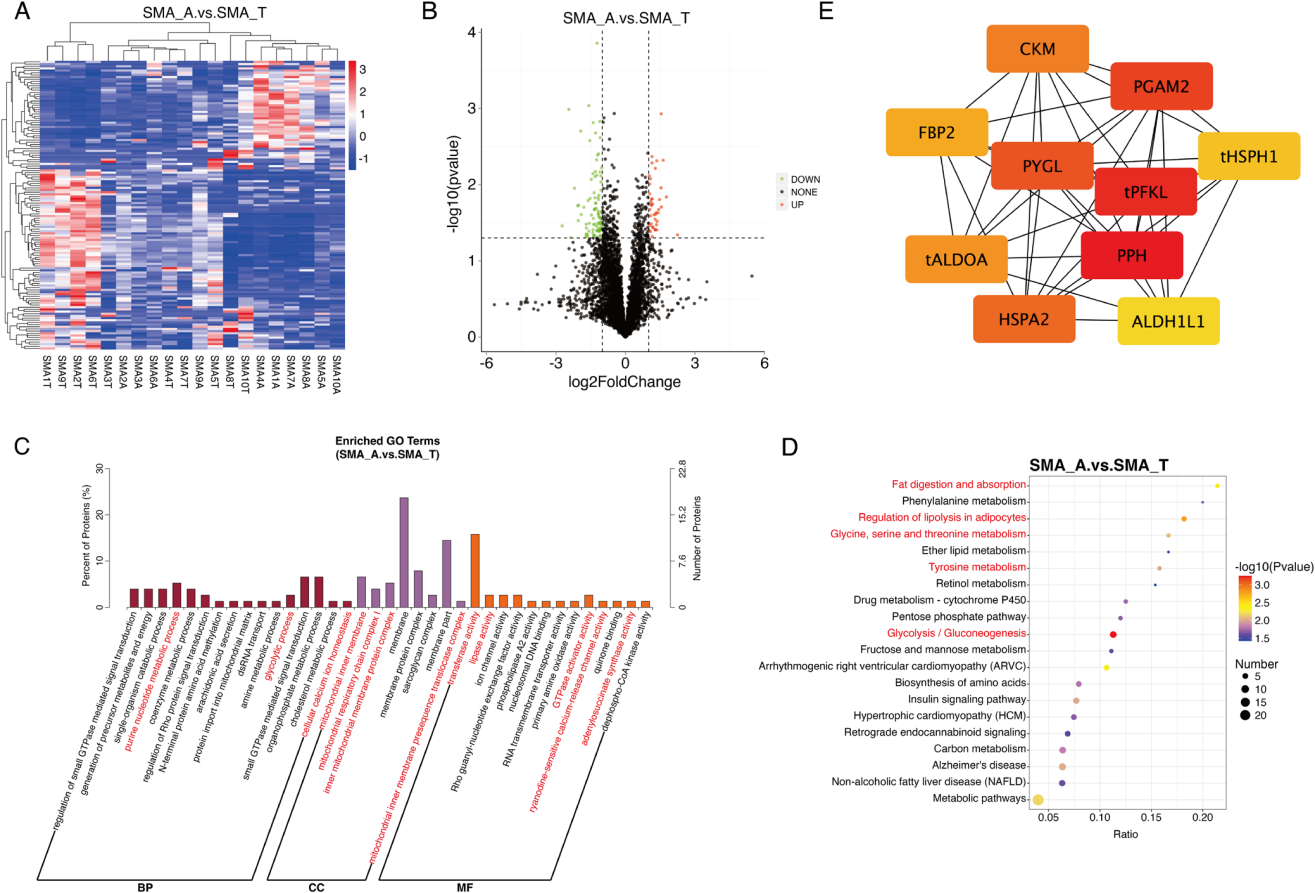
Proteomic comparison between concave and convex paraspinal muscles in SMA. (A, B) Heatmap and volcano plot of DEPs between the concave (SMA_A) and convex (SMA_T) sides. (C) Histogram of GO functional enrichment for DEPs. (D) Bubble plot of significantly enriched KEGG pathways for DEPs. (E) Top 10 hub proteins identified from the PPI network of DEPs between the two sides. SMA: Spinal muscular atrophy; SMA_A: Concave side; SMA_T: Convex side; DEP: Differentially expressed protein; GO: Gene ontology; KEGG: Kyoto Encyclopedia of Genes and Genomes; PPI: Protein–protein interaction.

A total of 133 DEPs were identified between SMA_A and SMA_T, with 41 upregulated in SMA_A and 92 in SMA_T (Figure 5A,B). GO enrichment analysis showed that these DEPs were enriched in BP including purine nucleotide metabolic process, glycolytic process, and cellular calcium ion homeostasis; CC including mitochondrial inner membrane, mitochondrial respiratory chain complex I, inner mitochondrial membrane protein complex, and mitochondrial inner membrane presequence translocase; and MF including transferase activity, GTPase activator activity, lipase activity, ryanodine‐sensitive calcium‐release channel activity, and adenylosuccinate synthase activity (Figure 5C). KEGG analysis revealed enrichment in glycolysis/gluconeogenesis, regulation of lipolysis in adipocytes, fat digestion and absorption, glycine‐serine–threonine metabolism, and tyrosine metabolism (Figure 5D). The top 10 hub proteins from the PPI network were PPH, truncated liver‐type phosphofructokinase (tPFKL), phosphoglycerate mutase 2 (PGAM2), heat shock‐related 70 kDa protein 2 (HSPA2), liver‐type glycogen phosphorylase (PYGL), creatine kinase M‐type (CKM), truncated fructose‐bisphosphate aldolase A (tALDOA), fructose‐1,6‐bisphosphatase isozyme 2 (FBP2), truncated 105 kDa heat shock protein (tHSPH1), and aldehyde dehydrogenase family 1 member L1 (ALDH1L1) (Figure 5E).

### 3.6 Metabolomic Profile of Paraspinal Muscles in SMA

**FIGURE 6:**
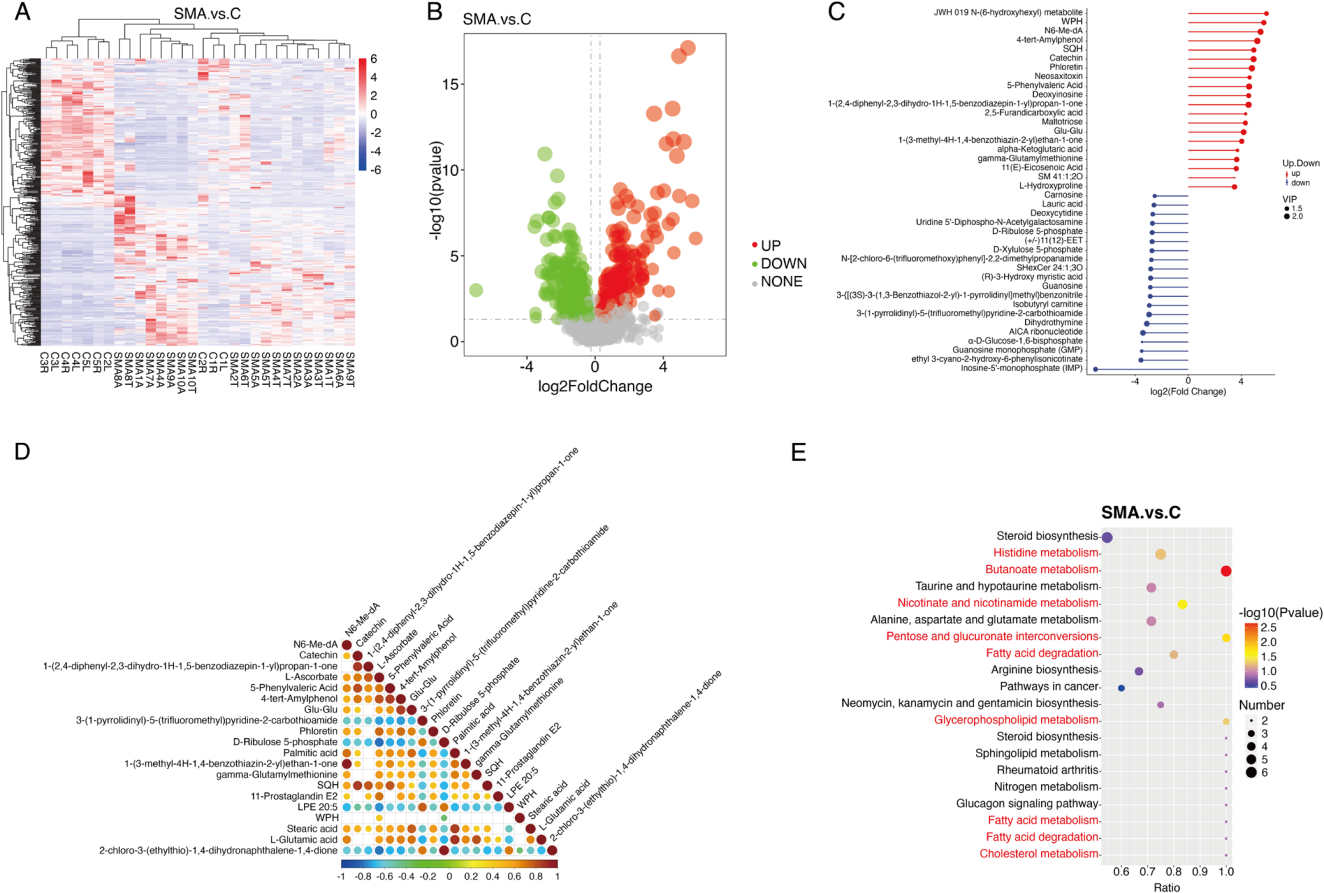
Metabolomic characteristics of paraspinal muscles in SMA. (A, B) Heatmap and volcano plot of DMs between the SMA and control groups. (C) Matchstick diagram illustrating the abundance changes of DMs. (D) Pearson correlation analysis of DMs. (E) Bubble plot of significantly enriched KEGG pathways for DMs. SMA: Spinal muscular atrophy; DM: Differential metabolite; KEGG: Kyoto Encyclopedia of Genes and Genomes.

Untargeted metabolomic analysis identified 370 DMs between SMA and control groups, with 179 upregulated and 191 downregulated in SMA (Figure 6A,B). Specifically, SMA muscles showed increased catechin, deoxycytidine, and α‐ketoglutaric acid, but decreased inosine‐5′‐monophosphate, guanosine monophosphate, and α‐D‐glucose‐1,6‐bisphosphate (Figure 6C). Correlation analysis revealed intricate metabolic interdependencies (Figure 6D). For instance, catechin levels showed strong positive correlations with 5‐phenylvaleric acid and L‐ascorbate, but were negatively associated with D‐ribulose 5‐phosphate and lyso‐phosphatidylethanolamine 20:5. Furthermore, D‐ribulose 5‐phosphate levels were inversely correlated with L‐ascorbate and 11‐prostaglandin E2. KEGG pathway enrichment highlighted global metabolic shifts, particularly in butanoate metabolism, pentose and glucuronate interconversions, and the metabolism of histidine, nicotinate, and nicotinamide. Additionally, fatty acid biosynthesis and glycerophospholipid metabolism were significantly altered (Figure 6E).

### 3.7 Metabolomic Divergence Between Bilateral Paraspinal Muscles in SMA

**FIGURE 7:**
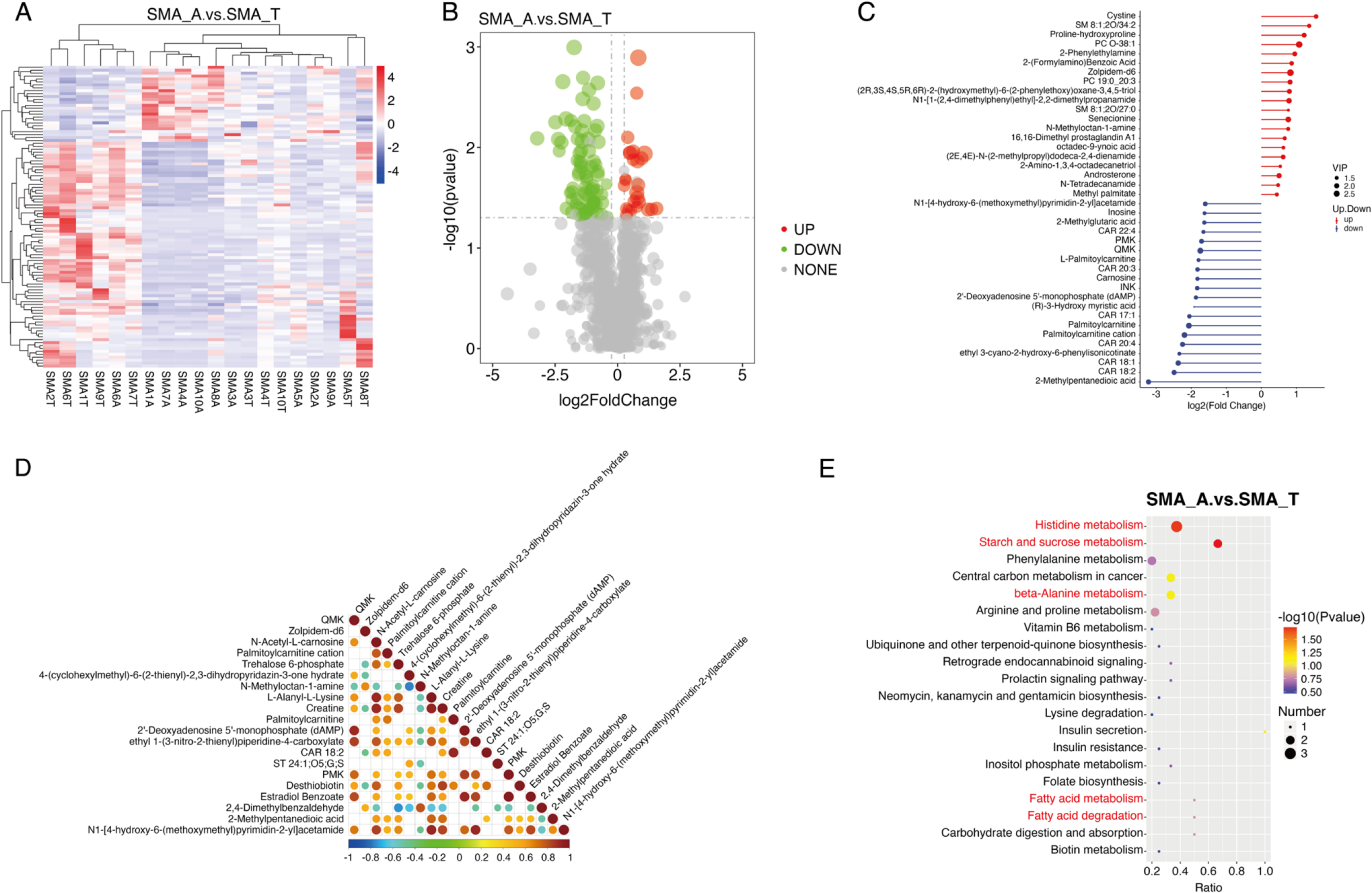
Metabolomic comparison of bilateral paraspinal muscles in SMA. (A, B) Heatmap and volcano plot of DMs between the concave (SMA_A) and convex (SMA_T) sides. (C) Matchstick diagram showing the distribution of DMs between sides. (D) Pearson correlation analysis of DMs. (E) Bubble plot of enriched KEGG pathways for DMs between the two sides. SMA: Spinal muscular atrophy; SMA_A: Concave side; SMA_T: Convex side; DM: Differential metabolite; KEGG: Kyoto Encyclopedia of Genes and Genomes.

Comparative metabolomics identified 94 DMs between SMA_A and SMA_T, with 17 upregulated in SMA_A and 77 in SMA_T (Figure 7A,B). SMA_A had elevated cystine, proline‐hydroxyproline, and androsterone, while SMA_T showed higher 2‐methylpentanedioic acid, palmitoylcarnitine, carnosine, and inosine (Figure 7C). Correlation analysis revealed specific metabolic clusters (Figure 7D). Creatine levels were positively associated with N‐acetyl‐L‐carnosine, palmitoylcarnitine, trehalose 6‐phosphate, L‐alanyl‐L‐lysine, dAMP, and estradiol benzoate. Notably, estradiol benzoate exhibited strong positive correlations with dAMP, L‐alanyl‐L‐lysine, and trehalose 6‐phosphate, suggesting a coordinated shift in localized hormone and energy metabolism. KEGG analysis highlighted that these DMs were predominantly involved in histidine, starch and sucrose, and β‐alanine metabolism (Figure 7E).

### 3.8 Integrated Multi‐Omics Signatures of Metabolic Dysregulation in SMA

**FIGURE 8:**
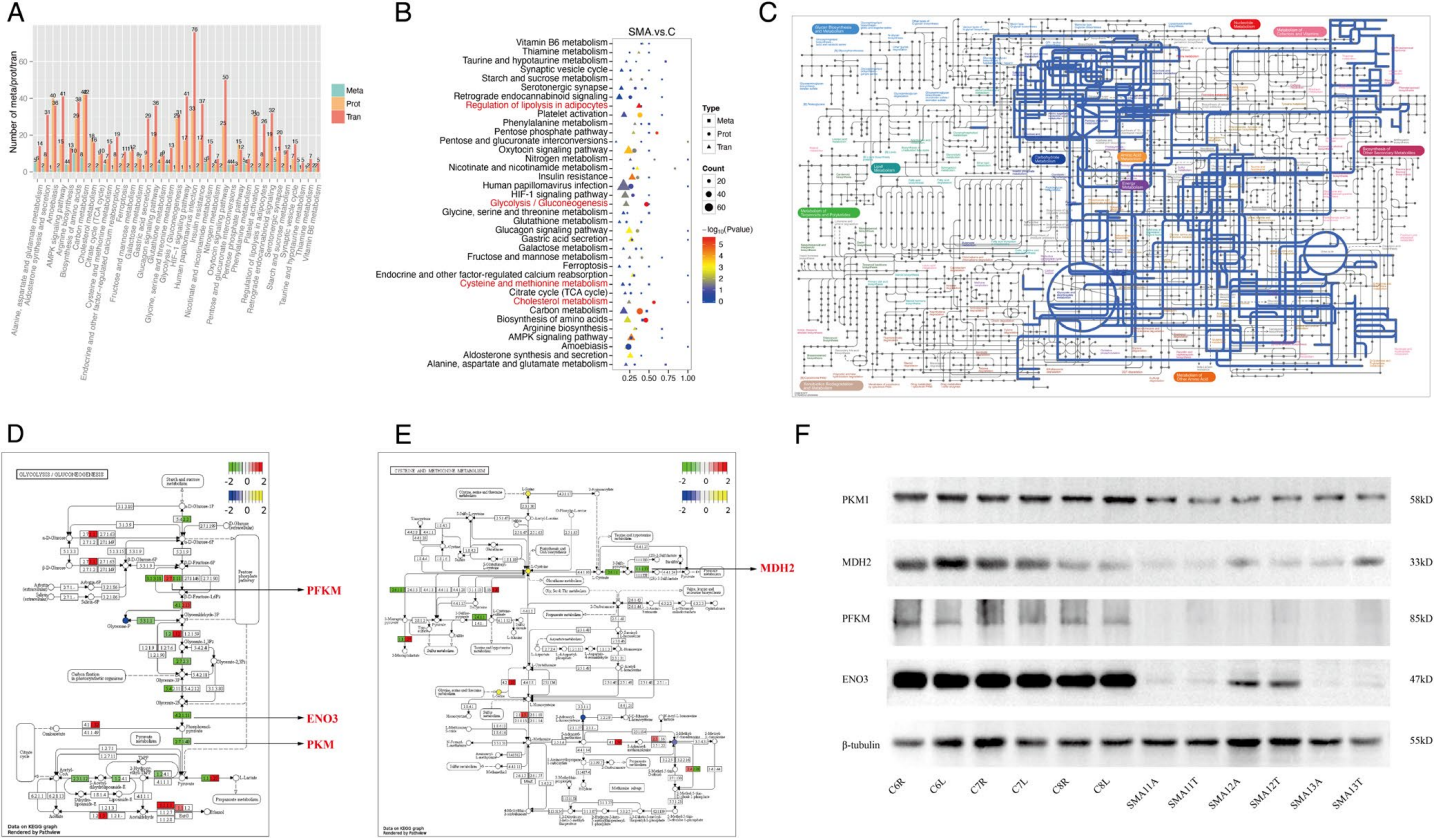
Integrated transcriptome, proteome and metabolome analysis comparing the control and SMA groups. (A, B) Histogram and bubble plots displaying enriched KEGG pathways and the distribution of DEGs, DEPs, and DMs within each pathway. (C) iPath analysis visualizing the global metabolic landscape of integrated omics data. (D, E) Detailed mapping of the Glycolysis/Gluconeogenesis and Cysteine/Methionine metabolism pathways. (F) Western blot validation of hub proteins within the PPI network involved in the aforementioned pivotal pathways. SMA: Spinal muscular atrophy; Tran: Differentially expressed gene; Prot: Differentially expressed protein; Meta: Differential metabolite; KEGG: Kyoto Encyclopedia of Genes and Genomes; iPath: Interactive pathways explorer; DEG: Differentially expressed gene; DEP: Differentially expressed protein; DM: Differential metabolite.

Integrated analysis identified 37 KEGG pathways concurrently dysregulated at transcriptomic, proteomic, and metabolomic levels in SMA, with 10 pathways related to carbohydrate metabolism and 7 involved in amino acid metabolism (Figure 8A,B). Complementary iPath analysis further confirmed systematic metabolic disturbances in carbohydrate and amino acid metabolism (Figure 8C). Within the glycolysis/gluconeogenesis pathway, we identified a signature of 29 DEPs, 31 DEGs, and 1 DM differentiating SMA from controls. Crucially, PPI network analysis pinpointed PFKM, PKM, and ENO3—key regulatory enzymes in these pathways—as top‐10 hub proteins (Figure 8A,D). MDH2 was similarly identified as a hub protein mediating cysteine and methionine metabolism (Figure 8A,E). These four metabolic hubs were subsequently validated by immunoblotting (Figure 8F) in an independent cohort (Table 1).

### 3.9 Integrated Multi‐Omics Landscape of Bilateral Paraspinal Muscle Asymmetry in SMA

**FIGURE 9:**
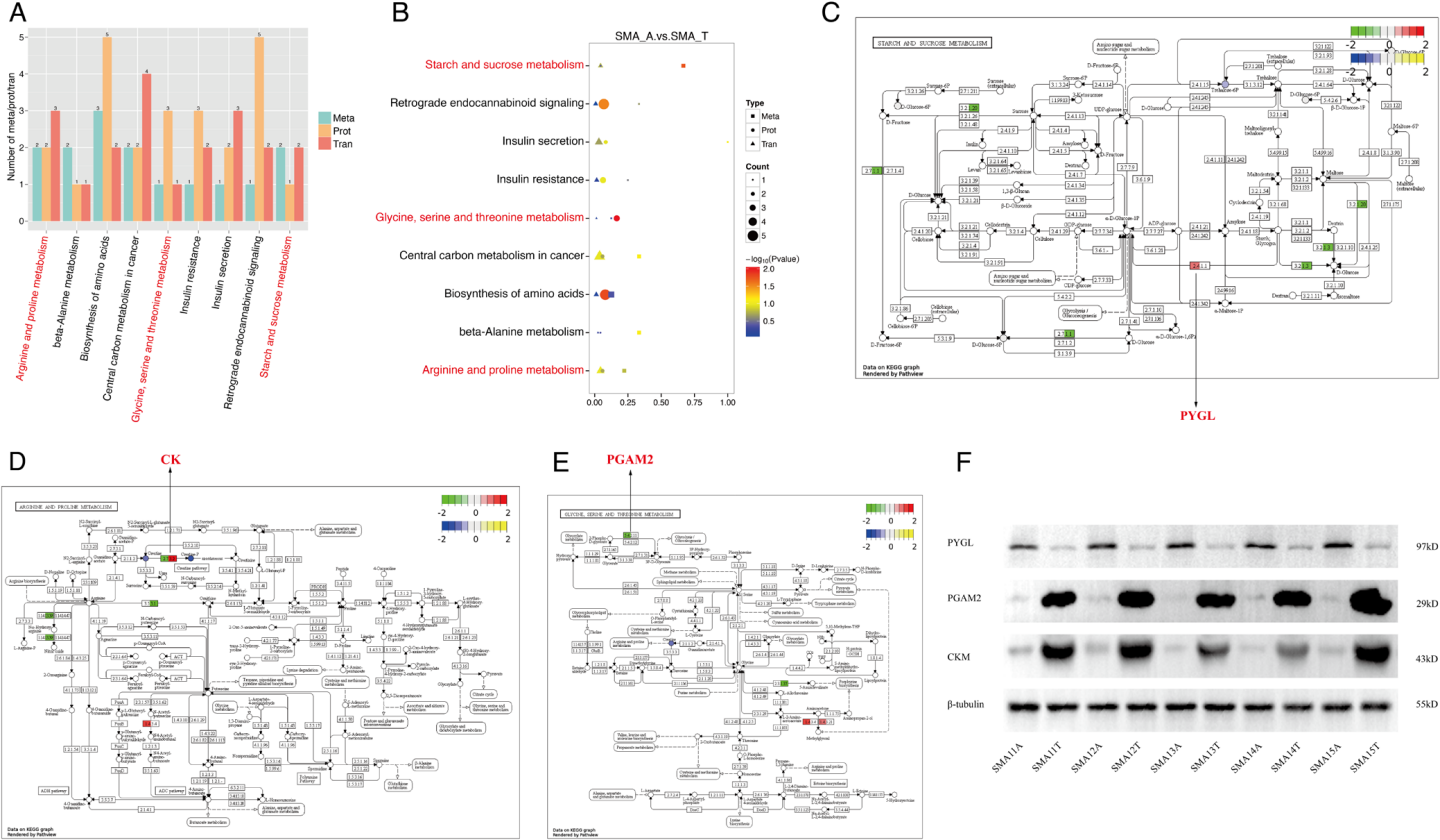
Integrated multi‐omics analysis of bilateral paraspinal muscles in SMA. (A, B) Histogram and bubble plots exhibiting enriched KEGG pathways and the constituent DEGs, DEPs, and DMs comparing the concave (SMA_A) and convex (SMA_T) sides. (C–E) Metabolic mapping of Starch/Sucrose, Arginine/Proline, and Glycine/Serine/Threonine metabolism pathways. (F) Western blot validation of hub proteins from the PPI network associated with these metabolic pathways between SMA_A and SMA_T groups. SMA: Spinal muscular atrophy; SMA_A: Concave side; SMA_T: Convex side; Tran: Differentially expressed gene; Prot: Differentially expressed protein; Meta: Differential metabolite; KEGG: Kyoto Encyclopedia of Genes and Genomes; DEG: Differentially expressed gene; DEP: Differentially expressed protein; DM: Differential metabolite.

Integrated analysis identified 9 KEGG pathways enriched with DEGs, DEPs, and DMs differentiating the SMA_A and SMA_T groups (Figure 9A). These pathways primarily converged on amino acid metabolism (e.g., glycine, serine, and threonine) and energy metabolism, such as starch and sucrose pathways (Figure 9B). PPI network analysis further prioritized three top‐10 hub proteins as key mediators of these metabolic shifts: PYGL, the sole DEP within the starch/sucrose pathway; CK, involved in arginine and proline metabolism; and PGAM2, which regulates glycine, serine, and threonine metabolism (Figure 9A,C–E). These hubs were validated by immunoblotting (Figure 9F) in an independent cohort of 5 SMA patients (Table 1), confirming asymmetric metabolic dysregulation.

## 4 Discussion

This study comprehensively characterizes SMA paraspinal muscles via multi‐omics, identifying distinct transcriptomic, proteomic, and metabolomic signatures. Key findings include significant carbohydrate and amino acid metabolic dysregulation in SMA muscle pathology and localized metabolic divergence between concave and convex sides linked to spinal deformity. These results define the molecular landscape of SMA muscle imbalance and identify potential metabolic hubs for further research.

SMA patients exhibit bilateral symmetric limb weakness with proximal predominance. Brogna et al. [8] further demonstrated that, in addition to the proximal‐to‐distal gradient of involvement, differential involvement also exists across muscle groups, characterized mainly by fatty infiltration and muscle atrophy. In the present study, transcriptomic analysis showed DEGs in paraspinal multifidus between SMA patients and controls were enriched in obesity, hyperlipidemia and adipocyte lipolysis‐related terms. Proteomic analysis revealed DEPs involved in lipid transport, cholesterol metabolism and PPAR signalling—a key regulator of adipocyte differentiation and lipid homeostasis. Metabolomic analysis identified DMs in fatty acid and cholesterol metabolism. Integrated multi‐omics consistently demonstrated dysregulated adipocyte lipolysis and cholesterol metabolism in SMA. These findings indicate systemic dysregulation of lipid transport, metabolism and degradation, as well as impaired adipocyte differentiation in SMA paraspinal muscles. Notably, while integrated multi‐omics analysis showed no enrichment in lipid metabolism–related pathways, transcriptomic analysis revealed asymmetric enrichment of DEGs between bilateral paraspinal muscles in adipocyte lipolysis regulation and lipid‐atherosclerosis pathways. Proteomic analysis confirmed DEP enrichment in adipocyte lipolysis regulation, fat digestion and lipase activity. Metabolomic changes were concentrated in fatty acid metabolism and degradation, consistent with transcriptomic asymmetry. Collectively, these data demonstrate asymmetric lipid metabolism dysregulation between concave and convex paraspinal muscles in SMA. Mouse models demonstrate that muscle weakness is an early event preceding overt motor neuron loss and muscle denervation [12]. Similarly, iPSC‐derived skeletal muscles from SMA patients exhibit reduced myotube size and impaired myogenic fusion [7]. Furthermore, in vitro studies have shown that SMN deficiency impairs myoblast fusion [13], collectively indicating that SMN plays a crucial role in myogenesis and muscle function beyond its role in motor neurons. In our study, DEGs between SMA patients and controls were enriched in GO terms for muscle development, myogenic differentiation, and muscle structure/function, as well as KEGG pathways including hypertrophic cardiomyopathy and cardiomyocyte adrenergic signalling. DEPs were similarly enriched in myosin/troponin complex terms and cardiomyopathy‐related pathways. These findings suggest a mixed atrophic and hypertrophic profile in SMA skeletal muscle, consistent with previous observations in type III SMA patients [6]. Of note, muscles in type III SMA are characterized by increased satellite cell populations and a shift toward fast‐twitch fibres. These cells overexpress genes associated with axonal guidance and neuromuscular junction remodelling, representing an active attempt to “re‐innervate” and restore functional connectivity.

In addition to the aforementioned KEGG pathways, DEGs between SMA patients and controls were enriched in energy metabolism–related pathways and calcium signalling pathways. Several hub genes from the DEG PPI network, such as GPER, MCHR1, and OPRD1, regulate the cAMP signalling pathway by modulating adenylate cyclase activity [14, 15, 16]. Significantly, these hub genes are also engaged in calcium ion transport [17, 18, 19, 20]. Consistently, DEPs were enriched in GO terms and KEGG pathways related to glucose metabolism and calcium ion binding and transport. Notably, the top 10 hub proteins in the DEP PPI network were all key regulators of carbohydrate metabolism, including glycolysis, the pentose phosphate pathway, and the citrate cycle. For example, PFKM, PKM, and ENO3 act as rate‐limiting glycolytic enzymes, while LDHA catalyses the final step of glycolysis. IDH2 and MDH2 participate in the citrate cycle, and TKT is essential for the non‐oxidative pentose phosphate pathway. Metabolomic analysis further identified DMs enriched in energy metabolism pathways such as glycolysis/gluconeogenesis and the pentose phosphate pathway. Integrated multi‐omics analysis confirmed disrupted energy metabolism in the paraspinal muscles of SMA patients. These abnormalities in energy metabolism and calcium handling may partly explain the enrichment of DEGs in muscle contraction–related GO terms. Multi‐omics analysis also revealed aberrant biosynthesis and metabolism of multiple amino acids in the paraspinal muscles of SMA patients. Most of these amino acids are critical for skeletal muscle structure, function, and development. For example, glycine promotes satellite cell proliferation and muscle mass while reducing muscle wasting [21, 22, 23, 24]; arginine enhances myogenic differentiation and myotube formation by increasing cytoplasmic calcium levels [25]; cysteine and methionine regulate muscle differentiation via glutathione synthesis [26]; and phenylalanine also facilitates myogenic differentiation [27]. Carbohydrate and amino acid metabolism may act synergistically in SMA, as several hub proteins are involved in both classes of metabolism. Among the top 10 hub proteins, TKT (a pentose phosphate pathway enzyme), IDH2 (citrate cycle), and glycolytic enzymes including PFKM, PKM, and ENO3 participate in amino acid biosynthesis, whereas MDH2 and LDHA are involved in cysteine and methionine metabolism. Of note, hub genes and proteins were selected based on network connectivity rather than on established causal evidence for the observed phenotypes. Accordingly, their pathogenic roles in SMA skeletal muscle warrant further experimental validation. The synergistic disturbances in carbohydrate and amino acid metabolism provide a mechanistic basis for the bioenergetic failure and mitochondrial dysfunction observed in SMA patients by Habets et al. [28]. Their magnetic resonance spectroscopy (MRS) findings of delayed inorganic phosphate recovery and blunted lactate responses might be explained by our identified molecular blocks: systemic dysregulation of glycolysis and amino acid metabolism. Combined impairment of glycolytic flux and amino acid anaplerosis limits mitochondrial substrate supply, thereby contributing to in vivo ATP deficiency. Our data also support the fibre‐type‐specific vulnerability and white‐to‐red myofiber shift reported in the MRS study, with glycolytic hub proteins (e.g., LDHA) significantly altered. Compromised primary and compensatory energy pathways further exacerbate fast‐twitch fibre dysfunction, which depends on tightly coupled glycolysis and amino acid metabolism. Consistently, several hub proteins we identified (PFKM, PKM, ENO3) are enriched in glycolysis/gluconeogenesis. Beyond its canonical role in spliceosome assembly and ribonucleoprotein biogenesis, SMN acts as a central regulator of bioenergetic metabolism. Specifically, SMN depletion impairs mitochondrial respiratory function, reduces ATP production, and disrupts the expression of key glycolytic enzymes (e.g., GAPDH, PGK1) by compromising the splicing, trafficking, or translation of metabolism‐related mRNAs [29]. Consistent with this mechanism, our identified DEPs are enriched in glycolysis/gluconeogenesis pathway, among which several hub proteins (e.g., PKM, ENO3) are precisely the downstream molecular targets of GAPDH. Beyond metabolic regulation, ultrastructural analysis indicate SMN deficiency induces focal and segmental alterations in the myofibrillar contractile apparatus [30]. Consistently, our transcriptomic analysis showed that 9 of the top 10 BP terms and 9 of the top 10 CC terms were muscle‐related, such as muscle contraction, myofibrils and sarcomeres, with DEGs including actin and troponin family genes. Similarly, proteomic analysis showed that DEPs between SMA and the control were enriched in CC terms, such as actin cytoskeleton, troponin complex and myosin complex.

In SMA, paraspinal muscle weakness stemming from neurogenic atrophy is recognized as a cardinal driver of spinal deformities. Conversely, the ensuing progression of scoliosis creates a detrimental feedback loop that further exacerbates muscular asymmetry. Consequently, elucidating the molecular and functional disparities between bilateral paraspinal muscles is imperative for the development of non‐surgical interventions aimed at re‐establishing muscle balance and attenuating scoliosis progression. GO and KEGG analyses of DEGs indicated asymmetric immune activation in the bilateral paraspinal muscles of SMA patients. Consistently, 7 of the top 10 hub genes in the PPI network (CCR1, CCR7, CXCL3, CCL20, RGS1, S1PR4, C5AR1) are involved in immune and inflammatory responses [31, 32, 33, 34, 35, 36, 37]. Notably, the differential enrichment of immune and inflammatory pathways by bulk RNA‐seq might reflect asymmetric infiltration of immune or inflammatory cells in bilateral paraspinal muscles, rather than differential gene expression in myocytes. Inflammation/immune pathways were differentially enriched between the concave and convex sides of paraspinal muscles in SMA patients, but not between SMA patients and controls. Despite rigorous inclusion criteria, potential localized inflammation in control paraspinal muscles might remain a confounding factor that may attenuate the enrichment of these pathways observed in SMA multi‐omics sequencing. DEGs in bilateral paraspinal muscles were also enriched in the calcium signalling pathway, supported by the finding that 8 of these 10 hub genes were linked to calcium ion transport. Similarly, DEPs showed divergent enrichment in cellular calcium ion homeostasis between the concave and convex paraspinal muscles. In addition, DEPs in bilateral paraspinal muscles of SMA were enriched in GO terms for glycolytic process and KEGG pathways for glycolysis/gluconeogenesis. Notably, key hub proteins involved in glucose metabolism within the bilateral paraspinal muscles appear to modulate the development and function of skeletal muscles in SMA. For example, PGAM2—which catalyses the conversion of 3‐phosphoglycerate to 2‐phosphoglycerate—supports muscle growth by promoting myoblast fusion [38]. Likewise, ALDOA is critical for muscle integrity; its loss causes rhabdomyolysis, and its serum elevation is a clinical hallmark of multiple myopathies [39, 40]. Notably, integrated multi‐omics analysis revealed asymmetric amino acid biosynthesis and metabolism in the bilateral paraspinal muscles of SMA patients. Several of these amino acids are critical for muscle function. For example, arginine and proline metabolism—regulated by the hub protein CKM—is essential for skeletal muscle health and regeneration [41]. The non‐proteogenic amino acid β‐alanine is the primary precursor for carnosine synthesis in skeletal muscle. Beyond its antioxidant and anti‐glycation properties, carnosine maintains muscle homeostasis by enhancing calcium sensitivity and contractility [42]. Notably, we observed an asymmetrical distribution of carnosine within the bilateral paraspinal muscles of SMA. Given the critical roles of these amino acids in muscle repair and physiology, we hypothesize that asymmetrical amino acid metabolism within the bilateral paraspinal muscles might contribute to the progression of SMA‐related scoliosis.

Similar to SMA, amyotrophic lateral sclerosis (ALS) is also characterized by motor neuron loss leading to progressive muscle atrophy. Transcriptomic profiling of ALS skeletal muscle reveals dysregulated genes enriched in muscle development, myoblast differentiation, and mitochondrial structure [43]. Furthermore, ALS‐associated gene expression patterns highlight disruptions in glycolysis and gluconeogenesis [44]. These findings are mirrored at the proteomic level, where dysregulated proteins participate in muscle development, muscle contraction and metabolic processes [45]. Notably, the secreted metabolome of ALS murine myocytes exhibits enrichment in pathways also perturbed in SMA, including arginine biosynthesis and nicotinamide metabolism [46]. Such multi‐omics parallels suggest that SMA and ALS share a common molecular substrate of myogenic decay. Duchenne muscular dystrophy (DMD) is an X‐linked disease driven by mutations in the dystrophin gene, leading to skeletal muscle atrophy. Dysregulated genes in skeletal muscles of DMD were mainly enriched in immune response and muscle regeneration [47]. Besides, proteomic analysis showed that DMD muscles exhibited dysregulation of proteins involved in glycolysis/gluconeogenesis, citrate cycle and amino acid metabolism [48]. Notably, a considerable portion of dysregulated metabolic proteins in DMD represent the same trend in SMA, indicating the similar metabolic disturbance between DMD and SMA. Consequently, SMA skeletal muscle appears to manifest a hybrid phenotype, encompassing characteristics of both neurogenic disorders and primary myopathies.

This study has several limitations. First, the high‐dimensional nature of multi‐omics data, contrasted with our limited sample size, poses inherent risks of statistical instability and false positives. In this discovery‐driven rare disease cohort, utilizing standard multiple‐testing corrections across comparisons might be overly conservative, particularly for the paired SMA samples, causing a high false‐negative rate that obliterates subtle pathological signals. To balance statistical power and false‐positive control, we employed a multi‐layered strategy—leveraging raw *p* values for discovery, while enforcing strict cross‐omics convergence, PPI hub constraints, and independent Western blot validation. Although target validation underscores our core conclusions, the broader omics landscape requires caution regarding exact effect sizes. Consequently, unconfirmed pathways like calcium signalling carry false‐positive risks and necessitate further experimental verification. Future studies with larger cohorts and advanced low‐sample statistical modelling are warranted to refine these findings. Second, the limited cohort and heterogeneous SMA phenotypes precluded definitive correlations of multi‐omics alterations with *SMN2* copy number, clinical subtype, or spinal deformity severity. A further limitation is the lack of concurrent radiological assessment during tissue sampling. To reduce redundant imaging, we utilized existing external MRI records in some patients; however, given the progressive nature of SMA, these historical scans likely misrepresent the muscle's metabolic state at surgery. Consequently, this temporal discrepancy prevented definitive clinicoradiological correlations. Larger patient cohorts are required to correlate multi‐omics data with clinical factors such as SMA subtype, paraspinal muscle radiology, and scoliosis severity. Notably, given distinct etiologies and biomechanics among SMA, IS and congenital scoliosis (CS), we used symmetrical spine controls and within‐patient convex–concave comparisons to avoid confounders; future studies may include IS/CS cohorts to validate scoliosis‐associated muscle signatures.

## 5 Conclusions

SMA is associated with abnormal carbohydrate and amino acid metabolism in paraspinal muscles. Furthermore, prominent metabolic asymmetries exist between the concave and convex sides of paraspinal muscles in these patients. These metabolic disturbances might be shaped by both SMN deficiency and the biomechanical stress of spinal deformity.

## Author Contributions

**Zhen Wang:** methodology, data curation, writing – original draft, conceptualization, visualization, writing – review and editing. **Xu'an Huang:** methodology, data curation, writing – review and editing, formal analysis. **Junduo Zhao:** writing – review and editing, methodology, visualization, software, formal analysis. **Jianxiong Shen:** conceptualization, funding acquisition, writing – review and editing, project administration, supervision. **Weiyun Chen:** conceptualization, project administration, writing – review and editing, supervision.

## Funding

This work was supported by National Natural Science Foundation of China, 82230083.

## Ethics Statement

This study was performed according to the Helsinki Declaration and approved by the Institutional Review Board of Peking Union Medical College Hospital (No. K4219).

## Consent

Written informed consent for participation was obtained from all adult patients or legal guardians.

## Conflicts of Interest

The authors declare no conflicts of interest.

## Data Availability

The raw transcriptomic data in this study have been deposited in the Genome Sequence Archive, China National Center for Bioinformation/Beijing Institute of Genomics, Chinese Academy of Sciences (https://ngdc.cncb.ac.cn/gsa‐human/) under the accession number HRA007820. The raw proteomic and metabolomic data have been deposited in the OMIX (https://ngdc.cncb.ac.cn/omix) under accession numbers OMIX006801 and OMIX006777, respectively.
